
JOURNAL INFORMATION
==============================
NLM Title Abbreviation: Wirtschaftsdienst
Journal ID: Wirtschaftsdienst
NLM Journal ID: 101086612

Wirtschaftsdienst (Hamburg, Germany : 1949)

ISSN: 0043-6275

ARTICLE INFORMATION
==============================
PMCID: PMC7237620
PMID: 32454541
DOI: 10.1007/s10273-020-2648-9
Article ID: 2648
Article version: 1
Subjects: Zeitgespräch

Globale Handelsordnung — mit den oder ohne die USA?

Diekmann Berend berend.diekmann@bmwi.bund.de

Referat VA1, Bundesministerin für Wirtschaft und Energie, Scharnhorststr. 34-37, 10115 Berlin, Deutschland
Electronic publication date: 2020 May 20
Print publication date: 2020
Volume: 100
Issue: 5
First page: 324
Last page: 328
Copyright: © Der/die Autor(en) 2020
License: Open Access Dieser Artikel wird unter der Creative Commons Namensnennung 4.0 International Lizenz veröffentlicht, welche die Nutzung, Vervielfältigung, Bearbeitung, Verbreitung und Wiedergabe in jeglichem Medium und Format erlaubt, sofern Sie den/die ursprünglichen Autor(en) und die Quelle ordnungsgemäß nennen, einen Link zur Creative Commons Lizenz beifügen und angeben, ob Änderungen vorgenommen wurden.
Die in diesem Artikel enthaltenen Bilder und sonstiges Drittmaterial unterliegen ebenfalls der genannten Creative Commons Lizenz, sofern sich aus der Abbildungslegende nichts anderes ergibt. Sofern das betreffende Material nicht unter der genannten Creative Commons Lizenz steht und die betreffende Handlung nicht nach gesetzlichen Vorschriften erlaubt ist, ist für die oben aufgeführten Weiterverwendungen des Materials die Einwilligung des jeweiligen Rechteinhabers einzuholen.
Weitere Details zur Lizenz entnehmen Sie bitte der Lizenzinformation auf http://creativecommons.org/licenses/by/4.0/deed.de.
License URL: https://creativecommons.org/licenses/by/4.0/

issue-copyright-statement: © The Author(s) 2020

==============================

Zur Ordnung des Welthandels haben die USA im 20. Jahrhundert stets eine zwiespältige Haltung eingenommen. Am Anfang einer protektionistischen Periode stand ein Zollgesetz, der Smoot-Hawley-Tariff-Act von 1930, der die US-Zölle für über 20.000 Produkte auf ein Rekordniveau anhob und den Außenhandel der USA innerhalb von drei Jahren um über 60 % zurückgehen ließ – mit desaströsen Folgen für die Weltwirtschaft. Ziel dieses protektionistischen Gesetzes war der Schutz der US-Wirtschaft vor ausländischer Konkurrenz. Alle Länder erhielten damals praktischen Anschauungsunterricht, zu welchen Ergebnissen eine exzessive Zollpolitik führen kann. Alle Länder, einschließlich der USA, lernten daraus. Gegen Ende des 2. Weltkriegs waren die USA der entscheidende Akteur bei der Gründung von Weltbank, Internationalem Währungsfonds (IWF) und später dem General Agreement on Tariffs and Trade (GATT). Die USA waren zu der Zeit das einzige große, industrialisierte Land mit intakten Produktionskapazitäten, einer modernen Kapitalausstattung und einer ausgebildeten Industriearbeiterschaft. Das überragende Interesse der USA an einer stabilen Weltwirtschaftsordnung mit offenen Märkten war also durchaus auch eigennützig: Nur stabile Märkte mit klaren Handelsregeln waren aufnahmebereit für US-Waren.

Mit wechselnden Motiven unterstützten die USA in den folgenden Dekaden die Weiterentwicklung der internationalen Handelsordnung bis hin zum Abschluss der Uruguay-Runde 1994, in der die Welthandelsorganisation (WTO) aus der Taufe gehoben wurde. Wesentliche Beweggründe waren die Idee, die ehemaligen Staatshandelsländer und etwas später auch das sich öffnende China in eine Welthandelsordnung zu integrieren, die von der Marktöffnung als Leitmotiv bestimmt war. Mit Sicherheit schwang dabei die Absicht mit, diese Märkte für Anbieter aus den USA besser zu öffnen und umgekehrt die USA selbst mit günstigen Zwischen- und Konsumprodukten zu versorgen. Diese Strategie verfolgten die USA ungeachtet des Umstands, dass das Verarbeitende Gewerbe der USA zu diesem Zeitpunkt im Außenhandel nur noch eine untergeordnete Rolle spielte, weil andere Länder, zuvörderst Japan, Deutschland und Südkorea, die USA hier überflügelt hatten.

Welchen Einfluss diese Entwicklung jedoch zugleich auf die Haltung der USA zum Freihandel ausüben konnte, war an der harten US-Reaktion auf die japanischen Exporterfolge in den 1980er Jahren abzulesen. Japanische Anbieter sahen sich einem enormen Druck ausgesetzt, der zu „freiwilligen" Exportbeschränkungen und mehr oder weniger erzwungenen japanischen Direktinvestitionen in den USA führte.

### Verschärfung der Konfliktlinien durch Verschiebung der weltwirtschaftlichen Gewichte …

Die Schwankungen in der US-Haltung zu einer multilateralen Handelsordnung haben sich nach 1994 sowohl durch interne wie durch externe Faktoren noch verstärkt. Zu den internen Faktoren zählten z.B. das Misstrauen gegenüber der Wirksamkeit von Regelsystemen im Welthandel und einer Beschränkung der eigenen Souveränität. Dieses Misstrauen war zwar schon immer vorhanden. Hinzu trat aber nun die verstärkte Wahrnehmung eines Gegensatzes zwischen Regeln und Resultaten. Die Regeln waren und sind aus US-Sicht entweder zu vage formuliert, die Sanktionsmechanismen zu langsam und unzureichend, sie sind für neue Herausforderungen (China) ungeeignet oder sie greifen zu sehr in die Souveränitätsrechte ein. Traditionell reagieren die USA zudem besonders empfindlich auf Konstruktionen, die dazu führen, dass internationale Entscheidungen US-Recht aushebeln können (Felbermayr, 2018). Als externe Faktoren kamen die Auslagerung von amerikanischer Produktion ins Ausland (offshoring), das Phänomen eines Wachstums ohne mehr Arbeitsplätze (jobless growth) und ein ausuferndes Handelsbilanzdefizit hinzu. Über allem rangierten der mit immer mehr Unbehagen wahrgenommene wirtschaftliche Aufstieg Chinas und das Scheitern der Integration Russlands in die Gruppe der marktwirtschaftlich und demokratisch verfassten Länder.

Diese Faktoren führten zu der Hinwendung der USA zu einer an schnelllebigen, vorteilhaften Resultaten orientierten Handelspolitik. Dies manifestiert sich nicht nur in klar definierten und inzwischen auch quantifizierten Marktzugangszielen der USA, wie schon beim von der Obama-Regierung formulierten Ziel der Verdopplung der US-Exporte zu beobachten. Vielmehr wuchs der Merkantilismus mit dem Amtsantritt der Trump-Administration in eine neue Dimension hinein. Einflussreiche Teile der neuen US-Regierung einschließlich des Präsidenten selber missverstehen die wirtschaftspolitische Aufgabe als betriebswirtschaftliches Thema und verkürzen Zusammenhänge: Sie denken in Ad-hoc-Deals, sehen ein Handelsbilanzdefizit als monetären Verlust und Handelsbilanzüberschüsse als Gewinn im betriebswirtschaftlichen Sinn. Sie beschränken sich auf die alleinige Betrachtung der Warenhandelsbilanz und blenden andere wichtige Zusammenhänge (wie z.B. den Handel mit Dienstleistungen, Primäreinkommen, Intrafirmenhandel) aus. Die schiere Größe des US-Markts bewegt sie dazu, Zölle bzw. generell politisches Wohlverhalten wie ein „Eintrittsgeld" in den US-Markt zu nutzen und die gestiegene geopolitische Unsicherheit bei kleineren Partnern merkantilistisch auszubeuten. In ihrem betriebswirtschaftlichen, kurzfristigen Kalkül gibt es sogar einen Anreiz, Unsicherheit zu schüren. Das resultatorientierte Verhalten führt zusammen mit einer betriebswirtschaftlichen Auffassung von Außenwirtschaft zu einem unsteten, um nicht zu sagen unzuverlässigen handelspolitischen Verhalten der USA.

Im transatlantischen Verhältnis ist zu beobachten, dass sich die USA auf Streitthemen konzentrieren, die durch asymmetrische Sensibilität gekennzeichnet sind, d.h. dort, wo die EU und ihre Mitgliedstaaten verwundbarer als die USA erscheinen (z.B. Autozölle, Leistungsbilanzüberschuss, Sekundärsanktionen), und dabei ihre dominante Rolle (z.B. US-Dollar als Leitwährung, New York als weltweit dominanter Finanzplatz, Technologieführerschaft, Größe des US-Markts) so unverblümt wie nie zuvor ausspielen. Die USA verknüpfen dabei gern Themen mit asymmetrischer Verwundbarkeit, zwischen denen keinerlei inhaltlicher Zusammenhang besteht, um so eine für sie noch vorteilhaftere Drohkulisse aufzubauen und der EU scheibchenweise Zugeständnisse abzupressen. Zugleich werben sie, wo von Vorteil, intensiv um Zusammenarbeit, ohne aber Konzessionen in anderen, strittigen Punkten auch nur anzudeuten.

Dieses Muster von Kooperation und Konfrontation lässt sich auch beim Streit um eine WTO-Reform beobachten. Im Mittelpunkt der US-Kritik an der WTO stehen die nicht mehr zeitgemäßen Regeln und der Streitbeilegungsmechanismus. Letzterem wird im Kern vorgeworfen, sein Auslegungsmandat zu überdehnen und das Handelsrecht durch seine Entscheidungen in einer Weise weiterentwickelt zu haben, die eigentlich Aufgabe von Verhandlungen gewesen wäre. Zugleich sei das WTO-Recht nicht geeignet, mit den chinesischen Handelspraktiken Schritt zu halten. Ferner seien Entscheidungsmechanismen ebenso wie Klassifizierungen vieler Länder als Entwicklungsländer und daraus resultierende Privilegien überholt. Allerdings nutzen die USA den von ihnen scharf kritisierten Streitbeilegungsmechanismus seit 1995 intensiv – ein Beleg für die Widersprüchlichkeit der US-Haltung. Von den in der WTO-Statistik zwischen 1995 und 2018 verzeichneten 595 Streitverfahren waren die USA in 124 Fällen

Beschwerdeführer und in 155 Fällen Beschwerdegegner (WTO, 2020; eigene Berechnungen). Damit entfielen auf die USA 47 % aller Fälle. Zusätzlich beteiligten sich die USA in 158 Fällen als Drittpartei. Folglich wird nur etwa ein Viertel aller Streitverfahren ohne US-Beteiligung geführt. In fast der Hälfte aller Verfahren traten die USA aktiv entweder als Beschwerdeführerin oder als Drittpartei auf. Besser lässt sich das rege Interesse der USA an der WTO-Streitbeilegung kaum belegen.

### … und die verhärtete innenpolitische Auseinandersetzung

Geradezu exemplarisch zeigt sich die – je nach politischer Ausrichtung der Regierung – stärker denn je schwankende US-Haltung zur Rolle des Freihandels insgesamt bei einem Vergleich der Argumentation in der Studie des Council of Economic Advisers (CEA) zu den wirtschaftlichen Vorteilen des Außenhandels von 2015 mit den Aussagen in der vom US-Handelsbeauftragten (USTR) 2018 erstellten Handelsagenda des Präsidenten (Council of Economic Advisers, 2015; United States Trade Representative, 2018): Der CEA stellte 2015 heraus, dass US-Unternehmen, die exportieren, durchweg höhere Löhne zahlen. Er verwies auf die dank des Handels um ein Viertel höhere Kaufkraft des durchschnittlichen US-Bürgers. Er betonte, dass Arbeitsstandards und Einkommen im Ausland dank des Handels erhöht würden, was wiederum gut für den Absatz US-amerikanischer Exporte sei. Der Umweltschutz profitiere, weil die Verbreitung moderner Technologien über Handel und Investitionen Umweltbelastungen überkompensiere. Handel verringere Diskriminierungen, weil im Sektor handelbarer Güter die Lohndifferenzierung zwischen männlichen und weiblichen Beschäftigten verringert würde und sich auch andere Arten von Diskriminierungen im Schnitt verringerten. Der CEA sprach 2015 von einem wertegetriebenen Handelsansatz (values driven trade approach), der die Kernanliegen der USA zu Umweltschutz, Nicht-Diskriminierung oder Arbeitsstandards aufnehme.

Ganz anders klingt es drei Jahre später in der Handelsagenda des Präsidenten. Dort werden fünf Leitlinien betont:

1. Stärkung der nationalen Sicherheit,

2. Stärkung der US-Wirtschaft,

3. Verhandlung besserer Handelsabkommen,

4. aggressive Durchsetzung von US-Handelsrecht,

5. Reform des multilateralen Handelssystems.

Insbesondere die betonte Rolle der nationalen Sicherheit für die Handelspolitik und die explizit angekündigte aggressive Durchsetzung von US-Handelsrecht akzentuieren den veränderten Ansatz. Aber auch hinter dem Ziel verbesserter Handelsabkommen verbirgt sich handelspolitischer Sprengstoff, denn spätestens seit dem Abschluss der Neuverhandlungen zum Nordamerikanischen Freihandelsabkommen (NAFTA), nunmehr US-Mexico-Canada Agreement (USMCA) genannt, ist klar, dass damit vor allem verschärfte Ursprungsregeln und mehr „local Content“ gemeint ist, also Bedingungen, die den globalen Handel nicht fördern, sondern bremsen. Von den nur drei Jahre zuvor vom CEA aufgelisteten Argumenten ist kaum noch etwas übrig.

### Welthandelsordnung fehlt zurzeit der Stabilitätsanker

Mit diesem handelspolitischen Ansatz können die USA die Rolle des kompensierenden Hegemons, die sie über Dekaden im GATT und zunächst auch in der WTO einnahmen, nicht mehr spielen. Hegemon sind die USA, weil sie über Jahrzehnte den Mitgliedern des GATT/der WTO nicht nur den Zugang zum lukrativen US-Markt zu stabilen Bedingungen boten, sondern auch ihr politisches Gewicht in die Waagschale warfen, damit die Regeln Glaubwürdigkeit und Gültigkeit behielten.

Allerdings hat die Welthandelsordnung mit dem protektionistischen Ansatz der USA nicht nur ihren Hegemon verloren. Man kann sie und die zugehörige Welthandelsorganisation WTO volkswirtschaftlich als Klubgut charakterisieren. Als Klubgut werden Güter bezeichnet, bei denen Ausschließbarkeit von der Nutzung möglich ist und keine oder eine nur geringe Rivalität im Konsum vorliegt. Die Mitgliedschaft in einem Klub ist freiwillig. Nicht-Zahler können von der Mitgliedschaft im Klub/der Nutzung des Klubguts ausgeschlossen werden. Diese Definition trifft grundsätzlich auf die WTO und ihre Mitgliedschaft zu. Allerdings funktioniert ein solches Klubgut umso besser, je homogener die Interessen der Klubmitglieder sind, je homogener die Leistungen ausfallen und je ausgewogener die Lastenverteilung ist (Langhammer, 2010). Die WTO-Mitglieder sind infolge der steigenden Mitgliederzahl inzwischen in ihren Interessen sehr heterogen. Auch die Homogenität der Themen ist angesichts der Ausweitung des Themenspektrums in der WTO nicht mehr gegeben. Um ein Extrembeispiel zu nehmen: Aus Zollverhandlungen im GATT sind zuletzt sogar Diskussionen um Handel und Geschlechtergerechtigkeit in der WTO geworden. Gerade für große WTO-Mitglieder ist es deshalb sehr attraktiv geworden, eine Homogenität des Klubgutes „Handelsordnung“ wieder herzustellen, indem sie ihre Interessen mit gleichgesinnten Partnern außerhalb der WTO verfolgen – z. B. durch bilaterale und plurilaterale Handelsabkommen.

Hier haben die USA sicher kein Alleinstellungsmerkmal, wie die zahllosen abgeschlossenen regionalen Abkommen insbesondere seit Gründung der WTO 1994 zeigen.

Damit gerät allerdings auch der originäre Anspruch der Welthandelsordnung ins Wanken: die Nicht-Diskriminierung auf Basis der Gegenseitigkeit. Sie besteht aus den beiden Komponenten Meistbegünstigung und Inländerbehandlung und ist damit so angelegt, dass auch Mitgliedsländer mit geringerer Marktgröße gleichberechtigt partizipieren können. Zusammen mit dem Gesamtverpflichtungsansatz (single undertaking), der es erlaubt, Marktöffnungen in unterschiedlichen Sektoren gegeneinander aufzurechnen, war die Handelsliberalisierung ursprünglich so strukturiert, dass alle Mitgliedstaaten ausreichend Spielräume für Erfolge und Zugeständnisse hatten. Da zugleich ein Hegemon vorhanden war, hatte das WTO-Regelwerk eine vergleichsweise hohe Bindungskraft und erschien nicht-kooperatives Verhalten lange Zeit unattraktiv.

In ihrer Distanzierung von der bisherigen Welthandelsordnung gehen die USA aber zuletzt konzeptionell auch noch deutlich über die eigenen Wege anderer großer WTO-Mitglieder wie der EU, die in ihren regionalen Abkommen durchaus eigene Akzente gesetzt hat, hinaus. Dies lässt sich gut am Beispiel des sogenannten Phase-I-Abkommens zwischen den USA und China belegen, das im Januar 2020 abgeschlossen wurde: Zwar wurde mit dem Abschluss dieses Teilabkommens eine weitere Eskalation des Handelskonflikts zwischen den zwei größten Volkswirtschaften der Welt erst einmal abgewendet. Gleichzeitig ist das Abkommen mit seinen umfassenden Kaufverpflichtungen für China aber Ausdruck eines kleinteiligen „Managed Trade“, der konzeptionell im scharfen Widerspruch zu den Grundsätzen des freien und regelbasierten Handels im Rahmen der WTO steht und im Zweifel zulasten von Drittländern geht. Anders als durch (Teil-) Substitution anderer Importe dürfte China insbesondere die Verpflichtungen im Agrarsektor, aber auch im Industriesektor, nicht erfüllen können.

Erschwerend kommt hinzu: Je höher der Anteil von Staatsunternehmen bzw. das Ausmaß staatlicher Lenkung in einem Sektor ist, desto leichter dürfte es China fallen, bestimmte quantitativ formulierte Einkaufszusagen zu erfüllen. Ein hoher Staatseinfluss ist in China beispielsweise im Energie-, Gesundheits- oder Luftfahrtsektor anzunehmen. De facto stärkt der US-Ansatz eines „Managed Trade“ mit China damit – paradoxerweise – den Staatshandelscharakter und die Rolle staatsnaher bzw. staatseigener Unternehmen in China.

Zu diesem geradezu planwirtschaftlichen Ansatz in der US-Handelspolitik tritt ein neues, kleinteiliges Buchstabieren des Reziprozitätsprinzips. Nach den Vorstellungen maßgeblicher US-Handelspolitiker soll sich die Reziprozität künftig nur noch auf die nominalen Zollsätze einzelner Produktlinien beziehen. Das käme einer vollständigen Aufgabe des Prinzips des Gesamtverpflichtungsansatzes (single undertaking) gleich. Als Hüter der bisherigen Welthandelsordnung kommen die USA, setzen sich diese Ansätze endgültig durch, nicht mehr infrage.

## Aber ohne die USA geht es nicht weiter

Damit steht insbesondere die europäische Handelspolitik vor einem Dilemma: Soll sie die – notwendige – Weiterentwicklung der Handelsordnung mit oder ohne die USA vorantreiben?

An dieser Stelle lohnt ein Blick in die Handelsdaten: Der Anteil der USA am Welthandel mit Waren ging von 22 % (1948) über 15 % (1960) und 12 % (1994) auf zuletzt 9 % (2018) zurück. Der Anteil der USA am Welthandel mit Dienstleistungen blieb seit 2005 mit 14 % in etwa konstant. Aus Deutschland gingen in die USA in den letzten zehn Jahren regelmäßig 8 % bis 9 % der Waren- und 15 % der Dienstleistungsexporte. Damit sind die USA der wichtigste Abnehmer deutscher Exporte. Noch zentraler ist die Rolle der USA als Standort für ausländische Direktinvestitionen: Der Anteil der USA am Weltbestand von ausländischen Direktinvestitionen liegt bei 28 % (2017). Für die Weiterentwicklung der internationalen Handelsordnung sind die Direktinvestitionen als Indikator wichtiger, weil sie näherungsweise den Intrafirmenhandel abbilden und damit eine bessere Näherungsgröße für die zukünftigen Schwerpunkte sowohl in regionalen Freihandelsabkommen als auch für eine globale Handelsagenda abbilden.

Damit lässt sich ein Fazit ziehen: Ungeachtet der inzwischen wankelmütigen bis feindseligen US-Haltung ist eine sinnvolle Weiterentwicklung einer globalen Handelsordnung ohne die USA kaum vorstellbar. Deshalb kann die Ersatz-Streitschlichtung, die die EU mit 15 anderen WTO-Mitgliedern Anfang April 2020 vereinbart hat, um die Lähmung des WTO-Berufungsgremiums durch die USA zu umgehen, auch nur als Provisorium, nicht als dauerhafte Alternative verstanden werden.

## Corona-Pandemie: Hoffnung auf eine neue Linie der USA?

Möglicherweise ändert die Corona-Epidemie die handelspolitischen Rahmenbedingungen. Der protektionistische Kurs der US-Handelspolitik, insbesondere die im Handelskonflikt mit China verhängten Zölle und die Drohungen gegenüber den wichtigsten US-Handelspartnern könnten dazu führen, dass die USA bei der Bekämpfung der

COVID-19-Pandemie massive Schwierigkeiten bekommen, Kranke zu versorgen – unter anderem weil medizinische Schutzausrüstung in den USA knapp wird. Vor dem Handelskonflikt mit China importierten die USA Medizinprodukte im Wert von ca. 5 Mrd. US-$ pro Jahr aus China (Bown, 2020). Das entspricht ca. einem Viertel der gesamten Importe von Medizinprodukten. China war unter anderem Hauptlieferant für Schutzausrüstung, die EU lieferte in erster Linie medizinisches Gerät wie z. B. Computertomografen oder Patientenmonitore. Durch die im transpazifischen Handelskonflikt verhängten US-Zölle auf chinesische Produkte erhöhten sich bisher niedrige oder nicht vorhandene Zölle auf Medizinprodukte massiv auf 15 % bzw. 25 %. Hierdurch verlagerte sich die US-Nachfrage nach Medizinprodukten: Die Importe aus China sanken zwischen 2017 und 2019 um 16 %. Importe aus dem Rest der Welt stiegen um 23 %.

Der US-Handelsbeauftragte (USTR) hat im März 2020 in Reaktion auf die Corona-Krise für bestimmte medizinische Produkte Ausnahmen von den Strafzöllen erteilt. Die Ausnahmen gelten rückwirkend zum 1. September 2019. Da die zusätzlichen Zölle für viele der jetzt rückwirkend von den Zöllen befreiten Produkte erst zum 1. September 2019 eingeführt worden waren, bedeutete die Entscheidung des USTR eine vollständige, der Not gehorchende Kehrtwende. Sie belegte zugleich eindrucksvoll die Fehlkalkulation der US-Handelspolitik, die die eigene Position (Unverwundbarkeit) überschätzte, was jetzt möglicherweise auch einer breiteren Öffentlichkeit bewusst wird und zu einer Gefahr für den Gesamtansatz der derzeitigen US-Handelspolitik werden könnte. Auch dürften die Effekte der Importerleichterungen begrenzt sein, da alle Länder – auch China – derzeit dazu neigen, den Export von medizinischen Schutzprodukten restriktiv zu handhaben und da ein halbes Jahr mit Zusatzzöllen ausgereicht haben dürfte, Handelsumlenkungen auszulösen, die nicht so schnell umkehrbar sind. Das generell gewachsene Misstrauen gegenüber der Sprunghaftigkeit der US-Handelspolitik könnte ein weiterer retardierender Faktor sein.

Dass diese Erfahrungen ausreichen, damit die USA ihren eigenen handelspolitischen Ansatz fundamental überdenken, erscheint im Lichte des US-typischen Pragmatismus möglich. Anstöße der wichtigsten US-Handelspartner zum richtigen Zeitpunkt, also unmittelbar nachdem das Schlimmste überwunden ist, könnten an der Stelle hilfreich wirken.

Dr. Berend Diekmann leitet das Referat USA, Kanada, Mexiko im Bundesministerium für Wirtschaft und Energie (BMWi) in Berlin.

Der Autor gibt seine persönliche Meinung wieder.


Literatur

Bown, C. (2020), Trump’s trade policy is hampering the US fight against COVID-19, https://www.piie.com/blogs/trade-investment-policy-watch/trump-trade-war-china-date-guide (17. April 2020).
Council of Economic Advisers (2015), The Economic Benefits of U. S. Trade, https://obamawhitehouse.archives.gov/sites/default/files/docs/cea_trade_report_final_non-embargoed_v2.pdf (17. April 2020).
Felbermayr G Zur Rückkehr der Machtpolitik in Handelsfragen: theoretische Überlegungen und politische Empfehlungen Perspektiven der Wirtschaftspolitik 2018 19 3 232 244 10.1515/pwp-2018-0027
Langhammer R Unordnung in der internationalen Handelsordnung: Befunde, Gründe, Auswirkungen und Therapien Perspektiven der Wirtschaftspolitik 2010 11 1 75 98 10.1111/j.1468-2516.2009.00322.x
United States Trade Representative (2018): President’s Trade Agenda, https://ustr.gov/about-us/policy-offices/press-office/reports-and-publications/2018/2018-trade-policy-agenda-and-2017 (17. April 2020).
Welthandelsorganisation WTO (2020), Disputes by Member, https://www.wto.org/english/tratop_e/dispu_e/dispu_by_country_e.htm#top (12. Mai 2020).
